# Relationship between maternal body mass index with the onset of breastfeeding and its associated problems: an online survey

**DOI:** 10.1186/s13006-020-00298-5

**Published:** 2020-06-15

**Authors:** Ana Ballesta-Castillejos, Juan Gomez-Salgado, Julian Rodriguez-Almagro, Inmaculada Ortiz-Esquinas, Antonio Hernandez-Martinez

**Affiliations:** 1 Department of Obstetrics & Gynaecology, Talavera de la Reina, 45600 Toledo, Spain; 2 Department of Sociology, Social Work and Public Health, University of Huelva, 21071 Huelva, Spain; 3 Safety and Health Postgraduate Programme, Universidad Espíritu Santo, 091650 Guayaquil, Ecuador; 4 Department of Nursing, Ciudad Real Nursing School, University of Castilla-La Mancha, 13071 Ciudad Real, Spain; 5 Department of Obstetrics & Gynaecology, Alcázar de San Juan, 13600 Ciudad Real, Spain

**Keywords:** Breastfeeding, Obesity, Public health

## Abstract

**Background:**

Obesity is a worldwide public health problem that demands significant attention. Several studies have found that maternal obesity has a negative effect on the duration of breastfeeding and delayed lactogenesis. The World Health Organization has classified Body Max Index (BMI) as normal weight (normoweight) (BMI:18.5–24.9), overweight (BMI:25–29.9), obesity grade I (30.0–34.9), obesity grade II (BMI: 35.0–39.9) and obesity grade III (BMI ≥ 40.0). The objective of this study is to describe the relationship between maternal BMI and breastfeeding rates, as well as breastfeeding-associated problems and discomfort in women assisted by the Spanish Health System.

**Methods:**

To this end, a cross-sectional observational study aimed at women who have been mothers between 2013 and 2018 in Spain was developed. The data was collected through an online survey of 54 items that was distributed through lactation associations and postpartum support groups between March and June 2019. Five thousand eight hundred seventy one women answered the survey. In the data analysis, Crude Odds Ratios (OR) and Adjusted Odds Ratios (AOR) were calculated through a multivariate analysis through binary and multinomial regression.

**Results:**

A linear relationship was observed between the highest BMI figures and the reduction of the probability of starting skin-to-skin contact (AOR for obesity type III of 0.51 [95% CI 0.32, 0.83]), breastfeeding in the first hour (AOR for obesity type III of 0.58 [95% CI 0.36, 0.94]), and exclusive breastfeeding to hospital discharge (AOR for obesity type III of 0.57 [95% CI 0.35, 0.94]), as compared to women with normoweight.

**Conclusions:**

Women with higher BMI are less likely to develop successful breastfeeding than women with normoweight.

## Background

Obesity, defined as an abnormal accumulation of fat that results in excessive body weight, is usually classified through the body mass index (BMI), defined as the weight in kilograms divided by the size in square meters (kg/m2) [1]. The World Health Organization (WHO) has classified BMI as normoweight (18.5–24.9), overweight (25–29.9), obesity grade I (30.0–34.5), obesity grade II (35.0–39.9), and obesity grade III (> 40.0) [2, 3].

Obesity is a worldwide public health problem that demands significant attention [4]. A recent study showed that the prevalence of obesity among pregnant women was 28.9%, a trend that continues to increase [4] globally among populations in both developing and developed countries [5]. According to the Perinatal European Health Report, in most European countries, more than 30% of pregnant women are obese. The proportion of overweight or obese women ranges from 30 to 50%, with a prevalence of less than 30% in Croatia, Austria, and Slovenia, and around 50% in the UK [6].

Recently, the relationship between BMI and exclusive maternal breastfeeding (EMB) has been identified as less common [7], and shorter [8] in women with a higher BMI. However, this association is not a novelty given that a study carried out in 1992 by Rutishauser and Carlin reported a 50% risk of early breastfeeding discontinuation among mothers with BMI > 26 kg/ m^2^ [4]. Due to the relevance of global obesity and overweight prevalence, numerous studies have focused on this issue and showed that maternal obesity has a negative effect on the duration of breastfeeding [9–11], as well as delayed lactogenesis [9, 12].

It is undeniable that obesity is an increasingly prevalent health problem. Specifically, in Spain, data indicate that the average population weight has increased since records exist, and if this trend continues [13], obesity rates will have increased by 16% by the year 2030 [14]. However, currently, there are no studies linking this data to newborn breastfeeding outcomes, so in this study we have considered describing the relationship between maternal BMI and breastfeeding rates, as well as breastfeeding-associated problems and discomfort in women assisted by the Spanish Health System.

## Methods

### Design and selection of the study participants

This is a cross-sectional observational study aimed at women who have been mothers between 2013 and 2018 in Spain. This study has received the approval of the Clinical Research Ethics Committee of Alcázar de San Juan, in Spain, with protocol number 92-C.

The participants were women over the age of 18, who understood Spanish and who agreed to fill in the questionnaire. Before filling in the questionnaire, the participants read the information sheet about the purpose of the study and give their consent to participate in it by ticking a box. After this, they were provided with the necessary information to be able to complete the questionnaire. Participants could voluntarily provide an email address or phone number through which they would be contacted in case any additional information related to the study was needed.

For the sample size estimation, the maximum modelling criterion that requires 10 events for each independent variable to be included in the multivariate model has been used [15]. For this sample calculation, the prevalence of delayed lactogenesis II has been used as the main event, which takes place in women with obesity around 58% [9]. Considering a minimum of 15 independent variables, a minimum of 150 women with delayed lactogenesis II would be required, representing a minimum total population of 259 women under study within this scenario. However, the research team decided to include all women who met the inclusion criteria in order to obtain estimates of greater precision.

For data collection, an anonymous online questionnaire was designed with 54 items [47 yes/no questions and 7 multiple answer questions) on sociodemographic and obstetric variables, and on complications during pregnancy, delivery, and postpartum (Additional file 1). The questionnaire was disseminated among the Spanish lactation and postpartum support associations and groups, and those responsible for these groups were in charge of disseminating them among their members (Additional file 2).

The variables included in the study were:

The main independent variable was self-reported Body Mass Index prior to gestation, categorised normoweight (18.5–24.9), overweight (25–29.9), obesity grade I (30.0–34.9), obesity grade II (35.0–39.9), and obesity grade III (≥ 40.0).

The independent adjustment variables were maternal age, income level, level of education, tobacco consumption, number of children, number of births, gestational age at the time of delivery, hypertension during pregnancy (high blood pressure during pregnancy), diet-controlled gestational diabetes (high sugar levels in blood during pregnancy, controlled with diet), insulin-controlled gestational diabetes (high sugar levels in blood during pregnancy, controlled with insulin), hyperthyroidism (high thyroid hormone levels), hypothyroidism (low thyroid hormone levels), anaemia (low iron levels in blood), intrahepatic cholestasis (reduction or obstruction of flow of bile in the liver), risk of preterm birth (risk of giving birth before week 37), deep vein thrombosis, oligohydramnios (decrease in amniotic fluid levels), polyhydramnios (increase in amniotic fluid levels), altered foetal heart rate (FHR) during delivery, stained amniotic fluid (AF), vaginal bleeding, non-progression of delivery, cephalopelvic disproportion, intrapartum fever, intrapartum preeclampsia, use of epidural analgesia, use of general anaesthesia, induced delivery, end of delivery, episiotomy, tearing, low birthweight (2.500 g), macrosomia (> 4.000 g), newborn admission, postpartum-related subsequent maternal surgery, maternal admission into intensive care unit, and maternal re-admission. The women were asked to mark the existence of a pathology only after having been diagnosed by a physician.

In addition, 3 “composite” morbidity variables were created: pregnancy (including Hypertension during pregnancy, diet-controlled gestational diabetes, insulin-controlled gestational diabetes, hyperthyroidism, hypothyroidism, anaemia, intrahepatic cholestasis, risk of preterm birth, deep vein thrombosis); delivery (including altered foetal heart rate (FHR) during delivery, stained amniotic fluid (AF), vaginal bleeding, non-progression of delivery, cephalopelvic disproportion, intrapartum fever (body temperature higher than 38 °C), intrapartum preeclampsia, induced delivery, end of delivery by caesarean, and severe tearing (type III-IV); neonatal (including prematurity, low birthweight (< 2.500 g), macrosomia (> 4.000 g), and neonatal admission to intensive care unit; and postpartum (including maternal postpartum surgery related to the delivery, maternal admission to intensive care unit, and maternal re-admission).

The dependent variables were: complications during lactation (starting skin-to-skin contact, starting breastfeeding in the first hour, and exclusive breastfeeding to hospital discharge), and complications or discomfort associated with breastfeeding during hospitalisation and at home.

### Statistical analysis

First, a descriptive analysis was performed using absolute and relative frequencies for the categorical variables. A bivariate analysis was then performed including the main sociodemographic, obstetric, neonatal, lactation and BMI characteristics at the time of delivery; this was done by using the Pearson’s chi-squared test with lineal trend. The analysis was performed to detect potentially confounding variables between the main dependent variables related to breastfeeding and BMI. In the next step, a multivariate analysis was carried out using binary logistic regression (through the ENTER procedure), where all the variables that could potentially be related to BMI were incorporated. Then, the Crude Odds Ratios (OR) and Adjusted Odds Ratios (AOR) were calculated with their respective 95% confidence intervals (95% CI) to determine the relationship between BMI and complications during breastfeeding (starting skin-to-skin contact, starting breastfeeding in the first hour, and exclusive breastfeeding to hospital discharge) and complications or discomfort both during hospitalisation and at home. All analyses were performed using the SPSS v24.0 statistical package.

## Results

The studied population was 5871 women. Of these, 27.3% (1607) had normoweight, 46.4% (2722) overweight, 19.6% (1153) obesity type I, 5.0% (293) obesity type II, and 0.13% (96) obesity type III. 56.1% (3126) were primiparous, 2.4% (142) had twin pregnancy, 6.9% (405) were preterm births (< 37 weeks), and 60.0% (3256) had an eutocic delivery.

A bivariate analysis was then performed to determine the sociodemographic, obstetric, neonatal, lactation and BMI characteristics at the time of delivery.

As for the relationship between BMI and the most important sociodemographic characteristics, a statistical association with maternal age, academic level, family income level, smoking habit, and attendance to childbirth was found. However, we did not find any statistically significant difference with respect to nationality and maternity classes. All the details of this analysis can be found in Table 1.

**Table 1:** Sociodemographic characteristics of the women according to their BMI

Variable	BMI						
	Normoweight *n* (%)	Overweight *n* (%)	Obesity type I *n* (%)	Obesity type II *n* (%)	Obesity type III n (%)	Total Values *n* (%)	*P -* value*
**Maternal age**							**< 0.001**
< 25 years	38 (2.4)	60 (2.2)	34 (2.9)	10 (3.4)	7 (7.3)	239 (4)	χ^2^ = 16.2
25–35	938 (58.4)	1545 (56.8)	697 (60.5)	192 (65.5)	66 (68.8)	3438 (57.6)	df = 1
> 35 years	631 (39.3)	1117 (41.0)	422 (36.6)	91 (31.1)	23 (24.0)	2284 (38.3)	
**Level of education**							**< 0.001**
None	0 (0.0)	4 (0.1)	2 (0.2)	2 (0.7)	0 (0.0)	8 (0.1)	χ^2^ = 99.9
Primary	28 (1.7)	48 (1.8)	21 (1.8)	6 (2.0)	3 (3.1)	106 (1.8)	df = 1
Secondary	340 (21.2)	715 (26.3)	379 (32.9)	132 (45.1)	45 (46.9)	1611 (27.4)	
University	1239 (77.1)	1955 (71.8)	751 (65.1)	153 (52.2)	48 (50.0)	4146 (70.6)	
**Monthly family income (euros)**							**< 0.001**
< 1000	81 (5.0)	139 (5.1)	74 (6.4)	30 (10.2)	9 (9.4)	333 (5.7)	χ^2^ = 69.4
1000–2000	469 (29.2)	891 (32.7)	416 (36.1)	128 (43.7)	45 (46.9)	1849 (32)	df = 1
2000–3000	571 (35.5)	924 (33.9)	397 (34.4)	83 (28.3)	29 (30.2)	2004 (34.7)	
3000–4000	331 (20.6)	533 (19.6)	202 (17.5)	41 (14.0)	9 (9.4)	1116 (19.3)	
> 4000	155 (9.6)	235 (8.6)	64 (5.6)	11 (3.8)	4 (4.2)	469 (8.1)	
**Nationality**							0.91
Spanish	1508 (93.8)	2557 (93.9)	1076 (93.3)	276 (94.2)	92 (95.8)	5509 (93.8)	χ^2^ = 0.01
Foreign	99 (6.2)	165 (6.1)	77 (6.7)	17 (5.8)	4 (4.2)	362 (6.2)	df = 1
**Attendance to maternal education**							0.36
No	375 (23.3)	602 (22.1)	271 (23.5)	61 (20.8)	35 (36.5)	1344 (22.8)	χ^2^ = 0.86
Yes	1232 (76.7)	2120 (77.9)	882 (76.5)	232 (79.2)	61 (63.5)	4527 (77.2)	df = 1
**Pre-pregnancy smoking habits**							**< 0.001**
No	1296 (80.6)	2104 (77.3)	834 (72.3)	201 (68.6)	69 (71.9)	4504 (76.7)	χ^2^ = 36.1
Yes	311 (19.4)	618 (22.7)	319 (27.7)	92 (31.4)	27 (28.1)	1367 (23.3)	df = 1

*Pearson’s χ^2^ test. df: degrees of freedom. Statistical significance at *p* ≤ 0.05

When analysing obstetric factors in relation to BMI, the relationship between BMI and the number of births, twin pregnancies, prematurity, hypertensive pregnancy disorders, gestational diabetes (both controlled with insulin and through diet), the risk of preterm birth, deep vein thrombosis, oligohydramnios, and polyhydramnios was analysed (Table 2).

**Table 2:** Obstetric characteristics of the women according to their BMI

Variable	BMI						
	Normoweight *n* (%)	Overweight *n* (%)	Obesity type I *n* (%)	Obesity type II *n* (%)	Obesity type III *n* (%)	Total values *n* (%)	*P* - value*
**Number of pregnancies**							**0.006**
One	860 (53.5)	1456 (53.5)	611 (53.0)	149 (50.9)	50 (52.1)	3126 (56.1)	χ^2^ = 7.4
Two	562 (35.0)	910 (33.4)	356 (30.9)	99 (33.8)	31 (32.3)	1658 (29.7)	df = 1
Three	137 (8.5)	232 (8.5)	120 (10.4)	33 (11.3)	11 (11.5)	533 (9.5)	
Four	36 (2.2)	84 (3.1)	39 (3.4)	8 (2.7)	2 (2.1)	169 (3)	
Five or more	12 (0.7)	40 (1.5)	27 (2.3)	4 (1.4)	2 (2.1)	85 (1.5)	
**Number of deliveries**							**< 0.001**
None	259 (16.1)	595 (21.9)	311 (27.0)	98 (33.4)	33 (34.4)	1296 (22.1)	χ^2^ = 40.0
One	869 (54.1)	1391 (51.1)	542 (47.0)	132 (45.1)	40 (41.7)	2974 (50.7)	df = 1
Two	422 (26.3)	645 (23.7)	261 (22.6)	50 (17.1)	20 (20.8)	1398 (23.8)	
Three	48 (3.0)	80 (2.9)	33 (2.9)	10 (3.4)	3 (3.1)	164 (2.8)	
Four	9 (0.6)	10 (0.4)	4 (0.3)	1 (0.3)	0 (0.0)	24 (0.4)	
Five or more	0 (0.0)	1 (0.0)	2 (0.2)	2 (0.7)	0 (0.0)	5 (0.0)	
**Twin pregnancy**							**0.004**
No	1583 (98.5)	2652 (97.4)	1118 (97.0)	284 (96.9)	92 (95.8)	5729 (97.6)	χ^2^ = 8.5
Yes	24 (1.5)	70 (2.6)	35 (3.0)	9 (3.1)	4 (4.2)	142 (2.4)	df = 1
**Prematurity**							**0.03**
> 37	1472 (91.6)	2557 (93.9)	1065 (92.4)	278 (94.9)	93 (96.9)	5465 (93.1)	χ^2^ = 4.5
< 37	135 (8.4)	165 (6.1)	88 (7.6)	15 (5.1)	3 (3.1)	406 (6.9)	df = 1
**Hypertensive states**							**< 0.001**
No	1550 (96.5)	2568 (94.3)	1032 (89.5)	239 (81.6)	77 (80.2)	5466 (93.1)	χ^2^ = 130.7
Yes	57 (3.5)	154 (5.7)	121 (10.5)	54 (18.4)	19 (19.8)	405 (6.9)	df = 1
**Gestational diabetes-diet**							**< 0.001**
No	1429 (88.9)	2463 (90.5)	988 (85.7)	223 (76.1)	75 (78.1)	5178 (88.2)	χ^2^ = 39.7
Yes	178 (11.1)	259 (9.5)	165 (14.3)	70 (23.9)	21 (21.9)	693 (11.8)	df = 1
**Gestational diabetes-insulin**							**< 0.001**
No	1575 (98.0)	2660 (97.7)	1124 (97.5)	275 (93.9)	89 (92.7)	5723 (97.5)	χ^2^ = 16.1
Yes	32 (2.0)	62 (2.3)	29 (2.5)	18 (6.1)	7 (7.3)	148 (2.5)	df = 1
**Hyperthyroidism**							0.34
No	1528 (95.1)	2601 (95.6)	1100 (95.4)	272 (92.8)	90 (93.8)	5591 (95.2)	χ^2^ = 0.9
Yes	79 (4.9)	121 (4.4)	53 (4.6)	21 (7.2)	6 (6.3)	280 (4.7)	df = 1
**Hypothyroidism**							0.43
No	1398 (87.0)	2375 (87.3)	984 (85.3)	251 (85.7)	86 (89.6)	5094 (86.7)	χ^2^ = 0.6
Yes	209 (13.0)	347 (12.7)	169 (14.7)	42 (14.3)	10 (10.4)	777 (13.3)	df = 1
**Anaemia**							**0.03**
No	967 (60.2)	1605 (59.0)	732 (63.5)	186 (63.5)	63 (65.6)	3553 (60.5)	χ^2^ = 4.5
Yes	640 (39.8)	1117 (41.0)	421 (36.5)	107 (36.5)	33 (34.4)	2318 (39.5)	df = 1
							0.50
No	1584 (98.6)	2691 (98.9)	1137 (98.6)	291 (99.3)	95 (99.0)	5798 (98.7)	χ^2^ = 0.5
Yes	23 (1.4)	31 (1.1)	16 (1.4)	2 (0.7)	1 (1.0)	73 (1.3)	df = 1
**Risk of preterm birth**							**0.003**
No	1433 (89.2)	2529 (92.9)	1062 (92.1)	276 (94.2)	87 (90.6)	5387 (91.7)	χ^2^ = 8.9
Yes	174 (10.8)	193 (7.1)	91 (7.9)	17 (5.8)	9 (9.4)	484 (8.2)	df = 1
**Deep vein thrombosis**							0.75
No	1580 (98.3)	2695 (99.0)	1138 (98.7)	289 (98.6)	92 (95.8)	5794 (98.6)	χ^2^ = 0.1
Yes	27 (1.7)	27 (1.0)	15 (1.3)	4 (1.4)	4 (4.2)	77 (1.3)	df = 1
**Oligohydramnios**							0.07
No	1561 (97.1)	2618 (96.2)	1092 (94.7)	285 (97.3)	93 (96.9)	5649 (96.2)	χ^2^ = 3.3
Yes	46 (2.9)	104 (3.8)	61 (5.3)	8 (2.7)	3 (3.1)	222 (3.8)	df = 1
**Polyhydramnios**							**0.001**
No	1502 (93.5)	2480 (91.1)	1037 (89.9)	259 (88.4)	82 (85.4)	5360 (91.3)	χ^2^ = 18.8
Yes	105 (6.5)	242 (8.9)	116 (10.1)	34 (11.6)	14 (14.6)	511 (8.7)	df = 1
**Composite pregnancy morbidity**							**0.02**
No	621 (38.6)	1051 (38.6)	419 (36.3)	93 (31.7)	31 (32.3)	2215 (37.7)	χ^2^ = 5.9
Yes	986 (61.4)	1671 (61.4)	734 (63.7)	200 (68.3)	65 (67.7)	3656 (62.3)	df = 1

*Pearson’s χ^2^ test. df: degrees of freedom. Statistical significance at *p* ≤ 0.05

Regarding complications during childbirth and postpartum period, there was a relationship between BMI and intrapartum fever, preeclampsia, induction of childbirth, type of delivery, presence of episiotomy, and birth composite morbidity (variable formed by the presence of all the complications during childbirth). No relationship was found with deep vein thrombosis, oligohydramnios, polyhydramnios, uterine rupture, general anaesthesia, grade III and IV perineal tear, postpartum surgery, maternal admittance to ICU, and maternal re-admittance to hospital. As for the newborn, children born to obese mothers were more likely to suffer from macrosomia and to be admitted to neonates’ units. Details of this analysis can be found in Table 3.

**Table 3:** Delivery and postpartum complications according to maternal BMI

Variable	BMI						
	Normoweight N (%)	Overweight n (%)	Obesity type I n (%)	Obesity type II n (%)	Obesity type III n (%)	Total values n (%)	*P* - value*
**Uterine rupture**							0.54
No	1595 (99.3)	2708 (99.5)	1148 (99.6)	288 (98.3)	95 (99.0)	5834 (99.3)	χ^2^ = 0.8
Yes	12 (0.7)	14 (0.5)	5 (0.4)	5 (1.7)	1 (1.0)	37 (0.7)	df = 1
**Fever**							**< 0.001**
No	1540 (95.8)	2574 (94.6)	1081 (93.8)	275 (93.9)	84 (87.5)	5554 (94.6)	χ^2^ = 12.1
Yes	67 (4.2)	148 (5.4)	72 (6.2)	18 (6.1)	12 (12.5)	317 (5.4)	df = 1
**Preeclampsia**							**< 0.001**
No	1577 (98.1)	2654 (97.5)	1103 (95.7)	266 (90.8)	85 (88.5)	5685 (96.8)	χ^2^ = 60.0
Yes	30 (1.9)	68 (2.5)	50 (4.3)	27 (9.2)	11 (11.5)	186 (3.2)	df = 1
**Epidural analgesia**							**< 0.001**
No	524 (32.6)	802 (29.5)	314 (27.2)	66 (22.5)	21 (21.9)	1727 (29.4)	χ^2^ = 19.4
Yes	1083 (67.4)	1920 (70.5)	839 (72.8)	227 (77.5)	75 (78.1)	4144 (70.6)	df = 1
**General anaesthesia**							0.09
No	1590 (98.9)	2692 (98.9)	1137 (98.6)	285 (97.3)	95 (97.3)	5799 (98.8)	χ^2^ = 2.86
Yes	17 (1.1)	30 (1.1)	16 (1.4)	8 (2.7)	1 (1.0)	72 (1.2)	df = 1
**Induced delivery**							**< 0.001**
No	1121 (69.8)	1708 (62.7)	688 (59.7)	151 (51.5)	52 (54.2)	3720 (63.3)	χ^2^ = 52.5
Yes	486 (30.2)	1014 (37.3)	465 (40.3)	142 (48.5)	44 (45.8)	2151 (36.7)	df = 1
**Mode of delivery**							**< 0.001**
Vaginal	1075 (66.9)	1629 (59.8)	639 (55.4)	136 (46.4)	47 (49.0)	3526 (60.0)	χ^2^ = 101.2
Instrumental	264 (16.4)	449 (16.5)	176 (15.3)	46 (15.7)	14 (14.6)	949 (16.1)	df = 1
Planned caesarean	89 (5.5)	220 (8.1)	108 (9.4)	41 (14.0)	6 (6.3)	464 (7.9)	
Emergency caesarean	179 (11.1)	424 (15.6)	230 (19.9)	70 (23.9)	29 (30.2)	935 (15.9)	
**Episiotomy**							**< 0.001**
No	1030 (64.1)	1812 (66.6)	822 (71.3)	218 (74.4)	74 (77.1)	3956 (67.4)	χ^2^ = 26.6
Yes	577 (35.9)	910 (33.4)	331 (28.7)	75 (25.6)	22 (22.9)	1915 (32.6)	df = 1
**III-IV tearing**							0.07
No	1589 (98.9)	2689 (98.8)	1136 (98.5)	284 (96.9)	95 (99.0)	5793 (98.7)	χ^2^ = 3.3
Yes	18 1.1	33 (1.2)	17 (1.5)	9 (3.1)	1 (1.0)	78 (1.3)	df = 1
**Composite morbidity delivery**							**< 0.001**
No	728 (45.3)	995 (36.6)	336 (29.1)	57 (19.5)	18 (18.8)	21.34 (36.7)	χ^2^ = 128.6
Yes	879 (54.7)	1727 (63.4)	817 (70.9)	236 (80.5)	78 (81.3)	3737 (63.3)	df = 1
**Postpartum surgery related to delivery**							0.10
No	1551 (96.5)	2634 (96.8)	1105 (95.8)	280 (95.6)	90 (93.8)	5660 (96.4)	χ^2^ = 2.6
Yes	56 (3.5)	88 (3.2)	48 (4.2)	13 (4.4)	6 (6.3)	211 (3.6)	df = 1
**Postpartum maternal ICU admission**							0.43
No	1575 (98.0)	2681 (98.5)	1128 (97.8)	285 (97.3)	94 (97.9)	5763 (98.2)	χ^2^ = 0.6
Yes	32 (2.0)	41 (1.5)	25 (2.2)	8 (2.7)	2 (2.1)	108 (1.83)	df = 1
**Readmission after discharge**							0.11
No	1580 (98.3)	2666 (97.9)	1132 (98.2)	281 (95.9)	94 (97.9)	5753 (98.0)	χ^2^ = 2.6
Yes	27 (1.7)	56 (2.1)	21 (1.8)	12 (4.1)	2 (2.1)	118 (2.0)	df = 1
**Composite postpartum morbidity**							0.08
No	1507 (93.8)	2558 (94.0)	1071 (92.9)	265 (90.4)	86 (89.6)	5487 (93.5)	χ^2^ = 5.6
Yes	100 (6.2)	164 (6.0)	82 (7.1)	28 (9.6)	10 (10.4)	384 (6.5)	df = 1
**Low birthweight**							0.13
No	1488 (92.7)	2560 (94.3)	1076 (93.4)	271 (93.1)	95 (99.0)	5490 (93.5)	χ^2^ = 2.3
Yes	118 (7.3)	155 (5.7)	76 (6.6)	20 (6.9)	1 (1.0)	370 (6.5)	df = 1
**Macrosomia**							**< 0.001**
No	1561 (97.2)	2608 (96.1)	1072 (93.1)	267 (91.8)	81 (84.4)	5589 (95.2)	χ^2^ = 56.4
Yes	45 (2.8)	107 (3.9)	80 (6.9)	24 (8.2)	15 (15.6)	271 (4.8)	df = 1
**Newborn admission**							**< 0.001**
No	1422 (88.5)	2399 (88.1)	991 (85.9)	231 (78.8)	82 (85.4)	5125 (87.3)	χ^2^ = 15.2
Yes	185 (11.5)	323 (11.9)	162 (14.1)	62 (21.2)	14 (14.6)	746 (12.7)	df = 1

*Pearson’s χ^2^ test. df: degrees of freedom. Statistical significance at *p* ≤ 0.05

Following this analysis (Table 4), we observed that skin-to-skin contact was performed in 77.9% of women (4574), the onset of breastfeeding after delivery in 74.5% (4372) of women, and 79.7% (4681) breastfed their baby at hospital discharge. When performing both the bivariate and the multivariate analyses, the three study variables were related with BMI. Thus, we observed that obese women experienced a lower rate of skin-to-skin contact at birth with an AOR of 0.79 for obesity type I (95% CI 0.65, 0.97), of 0.68 for obesity type II (95% CI 0.50, 0.93), and of 0.51 for obesity type III (95% CI 0.32, 0.83), as compared to mothers with normoweight. Lower rates of breastfeeding initiation were also observed in the first hour with an AOR of 0.80 for obesity type I (95% CI 0.66, 0.97), of 0.66 for obesity type II (95% CI 0.49, 0.90), and of 0.58 for obesity type III (95% CI 0.36, 0.94), as compared to mothers with normoweight. Likewise, a decrease in the probability of exclusive breastfeeding at discharge was observed for type III obese mothers, with an AOR of 0.57 (95% CI 0.35, 0.94), as compared to mothers with normoweight. These associations coincided when the analysis was performed by introducing the BMI as a quantitative variable.

**Table 4:** Breastfeeding complications according to the BMI

Variable	BMI					
	Normoweight ***n*** (%)	Overweight ***n*** (%)	Obesity type I ***n*** (%)	Obesity type II ***n*** (%)	Obesity type III ***n*** (%)	Total values ***n*** (%)
**Skin-to-skin contact**						
No	300 (18.7)	562 (20.6)	304 (26.4)	96 (32.8)	35 (36.5)	1297(22.1)
Yes	1307 (81.3)	2160 (79.4)	849 (73.6)	197 (67.2)	61 (63.5)	4574 (77.9)
OR CI 95	1 (ref.)	0.88 (0.76,1.03)	**0.64 (0.54, 0.77)**	**0.47 (0.36, 0.62)**	**0.40 (0.26, 0.62)**	
^a^AOR CI 95%	1 (ref.)	0.97 (0.82, 1.16)	**0.79 (0.65, 0.97)**	**0.68 (0.50, 0.93)**	**0.51 (0.32, 0.83)**	
^a^AOR CI 95% Total	**0.96 (0.95, 0.98)**					
**Start of breastfeeding in the first hour**						
No	358 (22.3)	661 (24.3)	339 (29.4)	106 (36.2)	35 (36.5)	1499 (25.5)
Yes	1249 (77.7)	2061 (75.7)	814 (70.6)	187 (63.8)	61 (63.5)	4372 (74.5)
OR CI 95%	1 (ref.)	0.89 (0.77, 1.04)	**0.69 (0.58, 0.82)**	**0.51 (0.39, 0.66)**	**0.50 (0.32, 0.77)**	
^b^AOR CI 95%	1 (ref.)	0.93 (0.79, 1.10)	**0.80 (0.66, 0.97)**	**0.66 (0.49, 0.90)**	**0.58 (0.36, 0.94)**	
^b^AOR CI 95% Total	**0.97 (0.95, 0.98)**					
**Exclusive breastfeeding at discharge**						
No	286 (17.8)	530 (19.5)	272 (23.6)	74 (25.3)	28 (29.2)	1190 (20.3)
Yes	1321 (82.2)	2192 (80.5)	881 (76.4)	219 (74.7)	68 (70.8)	4681 (79.7)
OR CI 95%	1 (ref.)	0.90 (0.76, 1.05)	**0.70 (0.58, 0.85)**	**0.64 (0.48, 0.86)**	**0.53 (0.33, 0.83)**	
^b^AOR CI 95%	1 (ref.)	0.91 (0.77, 1.08)	**0.79 (0.64, 0.97)**	0.78 (0.57, 1.07)	**0.57 (0.35, 0.94)**	
^b^AOR CI 95% Total	**0.96 (0.95–0.98)**					

For the multivariate analysis, the ENTER method for binary logistic regression was used

All variables adjusted by: maternal age, nulliparity, twin pregnancy, previous caesarean, smoking habit, economic income, level of education, nationality, maternity training, pregnancy composite morbidity, delivery composite morbidity, postpartum composite morbidity and epidural analgesia

^a^ Adjusted by: + prematurity

^b^Adjusted by: + newborn admission + prematurity + breastfeeding previous children

Pregnancy composite morbidity: including preeclampsia during pregnancy, diet-controlled gestational diabetes, insulin-controlled gestational diabetes, hyperthyroidism, hypothyroidism, anaemia, intrahepatic cholestasis, risk of preterm birth, deep vein thrombosis

Delivery composite morbidity: including altered foetal heart rate (FHR) during delivery, stained amniotic fluid (AF), vaginal bleeding, non-progression of delivery, cephalopelvic disproportion, intrapartum fever (body temperature higher than 38 °C), intrapartum preeclampsia, induced delivery, end of delivery by caesarean, and severe tearing (type III-IV)

Postpartum composite morbidity: including maternal postpartum surgery related to the delivery, maternal admission to intensive care unit, and maternal re-admission

Women were then asked about the different complications and/or problems that could have arisen throughout hospitalisation and at home after discharge. After the multivariate analysis, a link between maternal obesity in childbirth and various complications was observed. However, only a linear trend was observed during hospital admission between obesity and reduced breast engorgement, with an AOR of 0.70 for obesity type I (of 0.70) (95% CI 0.55, 0.89), of 0.63 for obesity type II (95% CI 0.42, 0.94), and of 0.42 for obesity type III (95% CI 0.19, 0.94), as compared to women with normoweight, as well as increased feelings of nervousness with an AOR of 1.23 for overweight women (of 1.23) (95% CI 1.06, 1.43), of 1.37 for obesity type I (95% CI 1.15, 1.64), of 1.60 for obesity type II (95% CI 1.20, 2.14), and of 1.69 for obesity type III (95% CI 1.05, 2.74), as compared to women with normoweight.

Once at home, the mothers also had lower breast engorgement with an AOR of 0.58 for obesity type II (95% CI 0.43, 0.77), and of 0.33 for obesity type III (95% CI 0.19, 0.58), an increased rise of delayed breast milk with an AOR of 1.31 for obesity type I (95% CI 1.01, 1.71), of 1.43 for obesity type II (95% CI 0.94, 2.16), and of 1.83 for obesity type III (95% CI 0.96, 3.50), as well as more problems related to the baby not gaining weight, with an AOR of 1.37 for obesity type II (95% CI 1.01, 1.84), and of 1.79 for obesity type III (95% CI 1.10, 2.90). When the analysis was again performed though including BMI as a quantitative variable, all the associations coincided except for difficulties with positioning the child due to C-section injury or episiotomy/tearing stitching, which showed a significant relationship. However, no relationship was found regarding baby not gaining weight. All the details of this analysis can be found in Table 5.

**Table 5:** Complications and discomfort during hospitalisation and at home related to breastfeeding according to maternal BMI

Variable	BMI					
Did you encounter any difficulty with breastfeeding while in hospital due to	Normoweight ***n*** (%)	Overweight ***n*** (%)	Obesity type I ***n*** (%)	Obesity type II ***n*** (%)	Obesity type III ***n*** (%)	Total values ***n*** (%)
**Pain?**						
No	902 (58.2)	1508 (57.8)	616 (55.9)	154 (55.6)	50 (58.8)	3230 (55.0)
Yes	647 (41.8)	1100 (42.2)	486 (44.1)	123 (44.4)	35 (41.2)	2391 (45.0)
OR CI 95	1 (ref.)	1.02 (0.90, 1.16)	1.10 (0.94, 1.29)	1.11 (0.86, 1.44)	0.98 (0.63, 1.52)	
^a^AOR CI 95%	1 (ref.)	1.01 (0.88, 1.15)	1.06 (0.90, 1.25)	1.03 (0.79, 1.35)	0.95 (0.60, 1.49)	
^a^AOR CI 95% Total	1.00 (0.99, 1.01)					
**Cracked nipples?**						
No	1092 (70.5)	1777 (68.1)	754 (68.5)	189 (68.2)	64 (74.4)	3876 (66.0)
Yes	457 (29.5)	831 (31.9)	346 (31.5)	88 (31.8)	22 (25.6)	1744 (34.0)
OR CI 95%	1 (ref.)	1.12 (0.98, 1.28)	1.10 (0.94, 1.29)	1.11 (0.85, 1.47)	0.82 (0.50, 1.35)	
^b^AOR CI 95%	1 (ref.)	1.11 (0.96, 1.27)	1.06 (0.89, 1.26)	1.05 (0.79, 1.39)	0.80 (0.48–1.32)	
^b^AOR CI 95% Total	1.00 (0.99, 1.01)					
**Difficulties for latching on to the breast/not proper latching on?**						
No	912 (59.2)	1481 (56.7)	575 (52.1)	134 (48.6)	51 (60.0)	3153 (53.7)
Yes	629 (40.8)	1131 (43.3)	528 (47.9)	142 (51.4)	34 (40.0)	2464 (46.3)
OR CI 95%	1 (ref.)	1.11 (0.98, 1.26)	**1.33 (1.14, 1.56)**	**1.54 (1.19, 1.99)**	0.97 (0.62, 1.51)	
^a^AOR CI 95%	1 (ref.)	1.11 (0.97, 1.27)	**1.27 (1.08, 1.50)**	**1.42 (1.08, 1.87)**	0.86 (0.54, 1.37)	
^a^AOR CI 95% Total	**1.02 (1.00, 1.03)**					
**Difficulties with positioning the child due to C-section injury or episiotomy/tearing stitching?**						
No	1290 (83.5)	2059 (79.0)	838 (76.0)	200 (73.0)	63 (73.3)	4450 (75.8)
Yes	254 (16.5)	547 (21.0)	265 (24.0)	74 (27.0)	23 (26.7)	1163 (24.2)
OR CI 95%	1 (ref.)	**1.35 (1.15, 1.59)**	**1.61 (1.32, 1.95)**	**1.88 (1.39, 2.53)**	**1.85 (1.13, 3.05)**	
^a^AOR CI 95%	1 (ref.)	**1.22 (1.03, 1.46)**	**1.29 (1.05, 1.58)**	1.32 (0.96, 1.82)	1.27 (0.75, 2.16)	
^a^AOR CI 95% Total	**1.02 (1.00, 1.04)**					
**Engorgement (swollen, tight, hard, hot breasts…)?**						
No	1312 (85.3)	2227 (85.7)	969 (88.3**)**	243 (88.7)	79 (91.9)	4830 (82.3)
Yes	227 (14.7)	373 (14.3)	129 (11.7)	31 (11.3)	7 (8.1)	767 (17.7)
OR CI 95%	1 (ref.)	0.97 (0.81, 1.16)	**0.77 (0.61, 0.97)**	0.74 (0.50, 1.10)	0.51 (0.23, 1.12)	
^a^AOR CI 95%	1 (ref.)	0.94 (0.78, 1.13)	**0.70 (0.55, 0.89)**	**0.63 (0.42, 0.94)**	**0.42 (0.19, 0.94)**	
^a^AOR CI 95% Total	**0.96 (0.94, 0.97)**					
**Your own nervousness?**						
No	1125 (72.9)	1790 (68.7)	713 (64.8)	165 (60.2)	51 (60.0)	3844 (68.6)
Yes	418 (27.1)	814 (31.3)	387 (35.2)	109 (39.8)	34 (40.0)	1762 (31.4)
OR CI 95%	1 (ref.)	**1.22 (1.07, 1.41)**	**1.46 (1.24, 1.73)**	**1.78 (1.36, 2.32)**	**1.79 (1.15, 2.81)**	
^a^AOR CI 95%	1 (ref.)	**1.23 (1.06, 1.43)**	**1.37 (1.15–1.64)**	**1.60 (1.20, 2.14)**	**1.69 (1.05, 2.74)**	
^a^AOR CI 95% Total	**1.03 (1.02, 1.05)**					
**Your feeling unsure of yourself?**						
No	987 (64.0)	1603 (61.5)	624 (56.6)	152 (55.3)	53 (62.4)	3419 (60.9)
Yes	556 (36.0)	1002 (38.5)	478 (43.4)	123 (44.7)	32 (37.6)	2191 (29.1)
OR CI 95%	1 (ref.)	1.11 (0.97, 1.26)	**1.36 (1.16, 1.59)**	**1.44 (1.11, 1.86)**	1.07 (0.68, 1.68)	
^a^AOR CI 95%	1 (ref.)	1.15 (0.99, 1.32)	**1.36 (1.15, 1.62)**	**1.44 (1.08, 1.91)**	1.09 (0.67, 1.79)	
^a^AOR CI 95% Total	**1.02 (1.01, 1.04)**					
**Did you encounter any difficulty with breastfeeding while at home due to**	**Normoweight** **n (%)**	**Overweight** **n (%)**	**Obesity type I** **n (%)**	**Obesity type II** **n (%)**	**Obesity type III** **n (%)**	**Total values** **n (%)**
**Pain?**						
No	746 (48.2)	1234 (47.3)	515 (46.8)	131 (48.0)	50 (58.8)	2676 (47.7)
Yes	801 (51.8)	1373 (52.7)	585 (53.2)	142 (52.0)	35 (41.2)	2936 (52.3)
OR CI 95%	1 (ref.)	1.04 (0.91, 1.19)	1.06 (0.91, 1.24)	1.01 (0.78, 1.31)	0.65 (0.42, 1.02)	
^b^AOR CI 95%	1 (ref.)	1.04 (0.92, 1.18)	1.04 (0.89, 1.22)	0.98 (0.75, 1.28)	0.67 (0.43, 1.06)	
^b^AOR CI 95% Total	0.99 (0.98, 1.01)					
**Cracked nipples?**						
No	915 (59.2)	1473 (56.6)	618 (56.3)	164 (60.7)	63 (74.1)	3233 (57.7)
Yes	631 (40.8)	1129 (43.4)	479 (43.7)	106 (39.3)	22 (25.9)	2367 (42.3)
OR CI 95%	1 (ref.)	1.11 (0.98, 1.26)	1.12 (0.96, 1.31)	0.94 (0.72, 1.22)	**0.51 (0.31, 0.83)**	
^b^AOR CI 95%	1 (ref.)	1.12 (0.98, 1.27)	1.12 (0.95, 1.31)	0.91 (0.69, 1.20)	**0.52 (0.31, 0.86)**	
^b^AOR CI 95% Total	0.99 (0.98, 1.01)					
**Difficulties for latching on to the breast/not proper latching on?**						
No	966 (62.6)	1662 (63.7)	639 (58.1)	150 (55.1)	55 (64.0)	3472 (61.8)
Yes	578 (37.4)	947 (36.3)	461 (41.9)	133 (44.9)	31 (36.0)	2150 (38.2)
OR CI 95%	1 (ref.)	0.95 (0.84, 1.09)	**1.21 (1.03, 1.41)**	**1.36 (1.05, 1.76)**	0.94 (0.60, 1.48)	
^b^AOR CI 95%	1 (ref.)	0.95 (0.83, 1.09)	1.16 (0.99, 1.37)	1.29 (0.98, 1.70)	0.88 (0.55, 1.41)	
^b^AOR CI 95% Total	1.01 (0.99, 1.03)					
**Difficulties with positioning the child due to C-section injury or episiotomy/tearing stitching?**						
No	1288 (83.2)	2094 (80.4)	862 (78.2)	211 (77.6)	69 (80.2)	4524 (80.6)
Yes	261 (16.8)	512 (19.6)	240 (21.8)	61 (22.4)	17 (19.8)	1091 (19.4)
OR CI 95%	1 (ref.)	**1.21 (1.02, 1.42)**	**1.37 (1.13, 1.67)**	**1.43 (1.04, 1.95)**	1.22 (0.70, 2.10)	
^b^AOR CI 95%	1 (ref.)	1.12 (0.95, 1.33)	1.17 (0.95, 1.43)	1.13 (0.81, 1.57)	0.93 (0.53, 1.65)	
^b^AOR CI 95% Total	**1.02 (1.00, 1.04)**					
**Engorgement (swollen, tight, hard, hot breasts…)?**						
No	930 (60.2)	1633 (62.6)	696 (63.3)	193 (71.2)	70 (81.4)	3522 (62.8)
Yes	614 (39.8)	975 (37.4)	403 (36.7)	78 (28.8)	16 (18.6)	2086 (37.2)
OR CI 95%	1 (ref.)	0.90 (0.80, 1.03)	0.88 (0.75, 1.03)	**0.61 (0.46, 0.81)**	**0.35 (0.20, 0.60)**	
^b^AOR CI 95%	1 (ref.)	0.90 (0.79, 1.02)	0.85 (0.72, 1.00)	**0.58 (0.43, 0.77)**	**0.33 (0.19, 0.58)**	
^b^AOR CI 95% Total	**0.96 (0.95, 0.98)**					
**Candidiasis (fungus)?**						
No	1486 (96.1)	2486 (95.6)	1035 (94.5)	263 (96.7)	83 (96.5)	5353 (95.6)
Yes	61 (3.9)	115 (4.4)	60 (5.5)	9 (3.3)	3 (3.5)	248 (4.4)
OR CI 95%	1 (ref.)	1.13 (0.82, 1.54)	1.41 (0.98, 2.04)	0.83 (0.41, 1.70)	0.88 (0.27, 2.87)	
^b^AOR CI 95%	1 (ref.)	1.10 (0.80, 1.52)	1.36 (0.94, 1.98)	0.75 (0.36, 1.54)	0.83 (0.25, 2.73)	
^b^AOR CI 95% Total	1.00 (0.97, 1.03)					
**Delayed milk production (after the 5th day of delivery)?**						
No	1406 (90.8)	2343 (89.9)	959 (87.2)	233 (86.0)	71 (82.6)	5012 (89.3)
Yes	142 (9.2)	262 (10.1)	141 (12.8)	38 (14.0)	15 (17.4)	598 (10.7)
OR CI 95%	1 (ref.)	1.11 (0.89, 1.37)	**1.46 (1.14, 1.86)**	**1.62 (1.10, 2.37)**	**2.09 (1.17, 3.75)**	
^b^AOR CI 95%	1 (ref.)	1.08 (0.86, 1.35)	**1.31 (1.01, 1.71)**	1.43 (0.94, 2.16)	1.83 (0.96, 3.50)	
^b^AOR CI 95% Total	**1.03 (1.01, 1.05)**					
**The baby not gaining enough weight?**						
No	1180 (76.1)	1976 (76.0)	847 (77.0)	188 (69.4)	57 (66.3)	4248 (75.7)
Yes	371 (23.9)	624 (24.0)	253 (23.0)	83 (30.6)	29 (33.7)	1360 (24.3)
OR CI 95%	1 (ref.)	1.00 (0.87, 1.16)	0.95 (0.79, 1.14)	**1.40 (1.06, 1.86)**	**1.62 (1.02, 2.57)**	
^b^AOR CI 95%	1 (ref.)	1.00 (0.86, 1.16)	0.93 (0.77, 1.13)	**1.37 (1.01, 1.84)**	**1.79 (1.10, 2.90)**	
^b^AOR CI 95% Total	1.01 (0.99, 1.03)					

For the multivariate analysis, the ENTER method for binary logistic regression was used

All variables adjusted by: Maternal age, nulliparity, twin pregnancy, previous caesarean, smoking habit, economic income, level of education, nationality, maternity training, pregnancy composite morbidity, delivery composite morbidity, postpartum composite morbidity, neonatal composite morbidity, and breastfeeding previous children

^a^Adjusted by: Breastfeeding first hour

^b^Adjusted by: Lactation type after hospital discharge

Pregnancy composite morbidity: including preeclampsia during pregnancy, diet-controlled gestational diabetes, insulin-controlled gestational diabetes, hyperthyroidism, hypothyroidism, anaemia, intrahepatic cholestasis, risk of preterm birth, deep vein thrombosis

Delivery composite morbidity: including altered foetal heart rate (FHR) during delivery, stained amniotic fluid (AF), vaginal bleeding, non-progression of delivery, cephalopelvic disproportion, intrapartum fever (body temperature higher than 38 °C), intrapartum preeclampsia, induced delivery, end of delivery by caesarean, and severe tearing (type III-IV)

Postpartum composite morbidity: including maternal postpartum surgery related to the delivery, maternal admission to intensive care unit, and maternal re-admission

Neonatal composite morbidity: including prematurity, low weight (< 2.500 g), macrosomia (> 4.000 g), and neonatal admission to intensive care unit

## Discussion

In this study we have investigated the relationship between BMI and the risk of complications during breastfeeding and its associated problems. Specifically, we have observed a linear relationship between higher BMI figures and a decrease in the likelihood of starting skin-to-skin contact, breastfeeding in the first hour, and exclusive breastfeeding at hospital discharge (Table 4). In addition, we also found a higher perception of lactogenesis delay and low weight gain in the newborn among the most important results (Table 5), coinciding with other authors [9, 12] that related BMI with late lactogenesis.

On the other hand, while this was not the study objective, we found what is in line with other research [6, 13, 16], that is an association between obesity and the risk of certain complications during pregnancy such as prematurity, hypertensive disorders in pregnancy, gestational diabetes (both controlled with insulin and through diet), risk of premature birth, deep vein thrombosis, oligohydramnios and polyhydramnios. Regarding complications during childbirth and postpartum, an increased risk of intrapartum fever [17], preeclampsia [6, 18], induction of delivery and caesarean delivery [6] was also observed. As for the newborn, children born to obese mothers were more likely suffer from macrosomy [6] and to be admitted to neonatal units [3, 19].

In this sense, our results coincide with those from other studies in which not only a lower likelihood of starting breastfeeding is observed [9–11], but also shorter duration if commenced [8]. Among the causes that may explain the lower probability of breastfeeding in obese women we find, on one hand, those related to all obstetric complications associated with obesity and, on the other, those related to differences attributable to a worse neuroendocrine response during the lactogenesis process. At the same time, other aspects of the psychosocial sphere [20] associated with obesity such as distress [10], increased risk of depression [10, 21], discrimination [22], worse body image [23, 24], social stigma [22], etc. may also influence.

Among the variables related to obstetric complications, the type of delivery stands out (Table 3). Obese women have an increased risk of C-sections [6, 25] and this is, in turn, associated with a greater premature separation of the mother-child binomial, thus hindering skin-to-skin contact and the onset of breastfeeding. However, we cannot make comparisons as no studies have been found that have delved into these aspects.

As for the neuroendocrine differences with respect to women with normoweight, there are several factors that can contribute to a delay in lactogenesis. First, it is known that decreased progesterone levels, which occurs after delivery, is one of the responsible factors for the preparation of the mammary gland [26]. Because progesterone is stored in adipose tissue, obese women may have more consistent hormone levels that may be responsible for inhibiting lactogenesis [4]. Second, another factor that could indirectly influence the perceived delay in lactogenesis is the anatomical characteristics of the obese woman’s breast, as adipose tissue between the ducts could affect the proper flow of milk [4]. Finally, there are certain disorders such as diabetes that, associated with maternal obesity, could interfere with the breastfeeding process. Women with diabetes and those who have a C-section may be more likely to experience delayed or low milk levels of lactogenesis [19]. These results could be explained by the lower prolactin concentration shown in obese mothers at rest and after the breastfeeding episode [4, 27]. Based on this fact and on our results, there would be several possible lines of action. On the one hand, as primary prevention measure, women should be recommended to reduce their BMI in pre-conception consultations so as to, among other benefits, attain successful breastfeeding. On the other hand, during breastfeeding, obese women should be encouraged to feed their children more often and that the intake takes longer, so as to stimulate milk production [28].

As for the limitations, a bias of information in considered unlikely as the data collected and the way in which the possible responses were presented did not require a high level of education. In this regard, the questions were asked in a basic and simple way, and any participant could understand them regardless of their level of education. It is not possible to completely rule out the confusion bias inherent in observational studies, but it has been controlled by using a multivariate analysis technique. As limitation, it is also worth mentioning that the proportion of foreign women and level of education is not representative of the whole present Spanish society, so these population groups were difficult to extrapolate. On the other hand, BMI was self-reported by women, and this was based on their clinical reports, according to the women’s own understanding of their clinical data, so there is a possibility that some women were not fully honest when giving their answers. However, several authors have observed that self-reported data on BMI can be successfully used in epidemiological studies as the differences are minimal among young populations and with a high level of education, as are the women in our sample [29–31]. Other limitations were the absence of underweight women among our sample and the impossibility of separating preeclampsia and chronic hypertension as different pathologies, as also happened with diabetes type I. Furthermore, the definition of the outcome variables such as hypertension or anaemia was based on the diagnosis recorded in the medical records and not on cut-off points of measurements or analytical values.

Among the main strengths of the study, the large sample size and the scarcity of studies focused on specific aspects of breastfeeding such as starting in the first hour or skin-to-skin contact can be highlighted. On the other hand, the prevalence of obesity in our study was similar to other studies, such as that by Parveen et al. [2], who observed a prevalence of obesity of 31.6%, and the one by Melchor et al. [32], with a prevalence of 18.4%. Because of this, we believe that our sample provides an adequate representation of women with high BMIs.

## Conclusions

Finally, we can conclude that women with higher BMI are more likely to give up breastfeeding and have problems associated with it. More specifically, these women are less likely to start skin-to-skin contact, breastfeeding within the first hour, exclusive breastfeeding at hospital discharge, and they also show a higher perception of late lactogenesis. As breastfeeding is considered a public health measure, mechanisms to support these women should be put in place both during their hospital admission and within the community. An approach to this situation from a qualitative point of view could be interesting so as to know more about the causes and enable the development of more effective intervention strategies.

## Supplementary information

**Additional file 1.** Questionnaire.

**Additional file 2.** List of associations that have collaborated in the distribution of the questionnaire.

## Data Availability

Not applicable.
