# Supplementary material for: Relationship between maternal body mass index with the onset of breastfeeding and its associated problems: an online survey
Source: Int Breastfeed J. 2020 Jun 15;15:55. doi: 10.1186/s13006-020-00298-5 (PMC7296910; doi:10.1186/s13006-020-00298-5)
Supplement: Supplementary file 1 — Additional file 1. Questionnaire. [file 13006_2020_298_MOESM1_ESM.docx]

**Additional file 1: QUESTIONNAIRE**

1. What is your age? (in years)

< 25 years

25-35 years

>35 years

2. What is your level of studies?

No studies

Primary

Secondary

University

3. Have you smoked before pregnancy?

No

Yes

4. Monthly family income (in euros)

<1000

1000-2000

2000-3000

3000-4000

>4000

5. Did you attend maternal education for your previous pregnancy?

No

Yes

6. What is your nationality?

Spanish

Foreign

7. How many pregnancies have you had?

One

Two

Three

Four

Five or more

8. How many vaginal deliveries have you had?

None

One

Two

Three

Four

Five or more

Now, we will ask you about your last pregnancy and delivery

**Important: You may only answer if the pathology has been diagnosed by a health professional and appears in your medical record.**

9. How many weeks were you pregnant when you gave birth?

Term (37 or more weeks)

Preterm (less than 37 weeks)

10. Was this a twin pregnancy?

No

Yes

11. During your last pregnancy, did you suffer from diagnosed high blood pressure?

No

Yes

12. During your last pregnancy, were you diagnosed with diabetes (high sugar levels) that was treated with diet?

No

Yes

13 During your last pregnancy, were you diagnosed with diabetes (high sugar levels) that was treated with insulin?

No

Yes

14. During your last pregnancy, were you diagnosed with hyperthyroidism?

No

Yes

15. During your last pregnancy, were you diagnosed with hypothyroidism?

No

Yes

16. During your last pregnancy, were you diagnosed with anaemia that was treated with iron?

No

Yes

17. During your last pregnancy, were you diagnosed with a liver disease (Intrahepatic cholestasis)?

No

Yes

18. During your last pregnancy, were you diagnosed with premature delivery (preterm) risk?

No

Yes

19. During your last pregnancy, were you diagnosed with venous thrombosis?

No

Yes

20. During your last pregnancy, were you diagnosed with low levels of amniotic fluid?

No

Yes

21. During your last pregnancy, were you diagnosed with high levels of amniotic fluid?

No

Yes

22. During your last delivery, did you have any problem due to diagnosed uterine rupture?

No

Yes

34. During your last delivery, did you have any problem due to fever, as assessed by a physician?

No

Yes

24. During your last delivery, did you have diagnosed high blood pressure (preeclampsia)?

No

Yes

25. Was it induced delivery?

No

Yes

67. How did your delivery end?

It was a normal or eutocic delivery

It was a forceps-aided delivery

It was a planned caesarean

It was an emergency caesarean

27. Did you have an episiotomy performed (a cut made by the midwife or the gynaecologist on the perineum during the delivery)?

No

Yes

28. Did you suffer any perineal tearing (other than episiotomy) that required suturing/stitches?

No

Yes

Yes, and later an appointment at hospital was scheduled for this reason (in this case, this would be a degree III-IV tearing, as these imply subsequent consultation follow-up).

29. Could you have skin-to-skin contact with your baby after the delivery?

No

Yes

30. Did you start maternal breastfeeding in the first hour following the delivery?

No

Yes

31. During your last pregnancy, did you give birth to a baby with weight < 2500 grams?

No

Yes

32. During your last pregnancy, did you give birth to a baby with weight > 4000 grams?

No

Yes

33. Was your child admitted to hospital ?

No

Yes

34. At discharge from hospital, was the newborn exclusively breastfed?

No

Yes

35. After the delivery, did you undergo any delivery-related surgery?

No

Yes

36. After the delivery, were you admitted at the ICU?

No

Yes

37. After discharge, were you admitted to hospital again?

No

Yes

38. Did you suffer any breastfeeding complication due to pain?

No

Yes

39. Did you suffer any breastfeeding complication due to tearing?

No

Yes

40. Did you suffer any breastfeeding complication due to difficulty with taking the breast?

No

Yes

41. While in hospital, did you suffer any breastfeeding complication due to diagnosed baby oral dysfunction (frenulum, mandibular retrognathism…)?

No

Yes

42. While in hospital, did you suffer any breastfeeding complication due to difficulties associated to posture caused by caesarean injury or episiotomy/tearing suturing?

No

Yes

43. While in hospital, did you suffer any breastfeeding complication due to engorgement (overfull, tense, hard, hot breasts…)?

No

Yes

44. While in hospital, did you suffer any breastfeeding complication due to nervousness?

No

Yes

45. While in hospital, did you suffer any breastfeeding complication due to insecurity?

No

Yes

46. While at home, did you suffer any breastfeeding complication due to pain?

No

Yes

47. While at home, did you suffer any breastfeeding complication due to tearing?

No

Yes

48. While at home, did you suffer any breastfeeding complication due to difficulties for the baby to take the breast?

No

Yes

49. While at home, did you suffer any breastfeeding complication due to diagnosed baby oral dysfunction (frenulum, mandibular retrognathism….)?

No

Yes

50. While at home, did you suffer any breastfeeding complication due to difficulties associated to posture caused by caesarean injury or episiotomy/tearing suturing?

No

Yes

51. While at home, did you suffer any breastfeeding complication due to engorgement (overfull, tense, hard, hot breasts…)?

No

Yes

52. While at home, did you suffer any breastfeeding complication due to candidiasis (fungal infection)?

No

Yes

53. While at home, did you suffer any breastfeeding complication due to late let-down of milk (later than the 5th day after delivery)?

No

Yes

54. While at home, did you suffer any breastfeeding complication due to diagnosed low weight gain in the baby?

No

Yes

What was your weight (in kg)? (State weight as measured by the health professional during the consultation)

What is your height (in cm)? (State height as measured by the health professional during the consultation)
