# Supplementary material for: Relationship between maternal body mass index with the onset of breastfeeding and its associated problems: an online survey
Source: Int Breastfeed J. 2020 Jun 15;15:55. doi: 10.1186/s13006-020-00298-5 (PMC7296910; doi:10.1186/s13006-020-00298-5)
Supplement: Supplementary file 2 — Additional file 2. List of associations that have collaborated in the distribution of the questionnaire. [file 13006_2020_298_MOESM2_ESM.docx]

**Additional file 2
List of associations that have collaborated in the distribution of the questionnaire**

Asociación “Dame Teta” de Albacete

Asociación de apoyo a la lactancia materna y crianza “La Rinconada”

Asociación Lactancia Melilla “Ca’mina”

Grupo de apoyo a la lactancia materna “Alma”

Grup Nodrissa de Denia

Asociación de apoyo a la lactancia materna de Elche

Asociación de mare a mare de Alcoy

La liga de la leche Comunidad Valenciana

Grupo de apoyo a la lactancia materna de Almería

Asociación de madres lactantes de Ávila

Asociación de lactancia materna Maire

Grupo de lactancia materna de Igualada

Grupo de lactancia materna de Hospital de Nens

Grupo de Apoyo a la lactancia materna de Osona

Grupo de apoyo a la lactancia materna  Areola

Federación Catalana de grupos de apoyo a la lactancia materna

Grupo de apoyo a la lactancia materna Mares d’orient

Colegio de Enfermería de Salamanca

Grupo de Lactancia y Crianza respetuosa  Amaryi de Cáceres

Grupo de Apoyo a la lactancia materna Regazo

Apoyo a la lactancia materna.  Mamare de Castellon

Grupo de Apoyo a la lactancia materna de Alcazar de San Juan

Asociación de lactancia materna de Tomelloso

Grupo de Apoyo a la lactancia materna de Ciudad Real

Grupo de Apoyo a la lactancia materna de Puertollano

Grupo de Apoyo a la lactancia “ALMamar”
Asociación Maternidad Compartida 
Grupo de Apoyo a la lactancia materna “Mamaré”
Grupo de Apoyo a la lactancia materna “Los Pedroches”

Asociación Madres de la Leche de Burgos 

Apoyo a la lactancia materna “Agambar”

Grupo de Apoyo a la lactancia materna “Dona a Dona”

Asociación de madres en lactancia y crianza de Palafrugell

Asociación Lactando “Amando”

Asociación Bastetana Luna de Leche

Asociación de Lactancia “Lactavida”

La liga de la leche de Euskadi

Grupo de Apoyo a la lactancia “Besoetan”

Grupo de Apoyo a la maternidad “Regazo Valverde”

Crianza Aljaraque
Asociación Balear de lactancia materna
La liga de la leche de Andalucía
Grupo de apoyo a la crianza y lactancia “Cazorlacta”
Asociación Nacer y Mamar
Lactancia Materna de Tomelloso
Grupo de Apoyo a la lactancia de León
Asociación de apoyo a la maternidad y lactancia natural “Lactabebé”
Asociación lleidatana prolactancia materna 
Grupo de Apoyo a la Lactancia “Boico de leite”
Grupo de refuerzo a la lactancia materna y la crianza
Asociación de apoyo a la lactancia materna “Entre nubes”
Grupo de Apoyo a la lactancia “Casa Grande de Tetuan”
Grupo de apoyo a la lactancia de madres a madres “Pantilla de mamás”
Asociación de Lactancia materna de Madrid “LacMad”

Asociación madres de la leche
La Liga de La Leche de Madrid
Asociación despertares Maternidad
Consulta de lactancia materna “Multilacta”
Asociación de lactancia materna “Parlacta”

Grupo de apoyo a la lactancia y crianza con apego del Corredor del Henares
Grupo de lactancia materna de Torrejón
Grupo de Apoyo a la lactancia materna “Lactamor”
Asociación de Crianza de Leganés
Asociación de Apoyo a la Lactancia y la Crianza con Apego de Leganés “Getalma”

Grupo de Lactancia, alimentación complementaria y crianza “Mamá Capaz”
Grupo de Apoyo a la lactancia “Rincón”
Grupo de Apoyo a la lactancia “Criar con apego”
Grupo de Apoyo a la Lactancia y Maternidad “Amagintza”
Grupo de Apoyo a la lactancia “De nai a nai”
Grupo de apoyo a la lactancia “Hermanas de leche” de Palencia

Asociación de Familias Enlazadas por una crianza saludable
La Liga de la Leche de Segovia 
Grupo de Apoyo Colectivo “La Leche”

Grupo de Apoyo a la lactancia “Lactarrinconada”
Asociación “Alletar i Criar”

Grupo de Apoyo a la lactancia de la Gomera
Grupo de Apoyo a la lactancia “MELIC”
Grupo de Apoyo a la lactancia materna “Amamanta”
Asociación para la normalización de la lactancia materna “Mammalia”

Grupo de Apoyo a la lactancia “La Safor Al Pit”
Asociación de Apoyo a la lactancia materna y crianza respetuosa “SINA”
Asociación Criar sin manual

Grupo de Apoyo a la Lactancia “Lactancia en positivo”
Grupo de Apoyo Postparto “Red de Mar”
La Liga de la Leche de Valladolid
Grupo de Apoyo a la lactancia Asociación “En nuestro Regazo”
Grupo de Apoyo a la lactancia “Creciento juntos”
Grupo de Apoyo a la lactancia “Apego Lacteo”
Asociación aragonesa de apoyo a la lactancia materna
Asociación Andaluza de Matronas
Asociación de Comares de la Comunitat Valenciana
Asociación Catalana de Llevadores
Asociación de Matronas de Madrid

Asociación de Matronas de Murcia
Asociación Balear Comares 
Asociación Científica de Matronas de Castilla-La Mancha
Asociación de Matronas Extremeñas
Asociación de Matronas de La Rioja
Asociación Gallega de Matronas
Asociación profesional de Matronas de Asturias
Asociación Canaria de Matronas
Asociación Científica de Matronas de Aragón

Asociación Cántabra de Matronas,
Asociación Nacional de Matronas

Asociación de Enfermería Pediátrica
Colegio de enfermería de Cantabria

Asociación de apoyo a la lactancia “Teta e Coliño”
